# Crescentic glomerulonephritis with anti-GBM antibody but no glomerular deposition

**DOI:** 10.1186/s12882-018-1027-x

**Published:** 2018-09-12

**Authors:** Omid Sadeghi-Alavijeh, Scott Henderson, Paul Bass, Terence Cook, Kirsten DeGroot, Alan David Salama

**Affiliations:** 1 UCL Centre for Nephrology, Royal Free Hospital, London, NW3 2PF UK; 2 Centre for Complement and Inflammation Research, Division of Medicine, Imperial College London, London, UK; 3 III rd Medical Department (Internal Medicine, Nephrology, Rheumatology) Sana Klinikum Offenbach, Offenbach, Germany

**Keywords:** Goodpasture’s disease, Anti- glomerular basement membrane antibodies, ANCA, Linear binding, Glomerulonephritis

## Abstract

**Background:**

Anti-glomerular basement membrane (GBM) antibodies are highly specific for Goodpasture’s or anti-GBM disease, in which they are generally directed against the non-collagenous (NC1) domain of the alpha 3 chain of type IV collagen(α3(IV)), and less commonly, toward the α 4(IV) or α 5(IV) chains, which form a triple helical structure in GBM and alveolar basement membrane (ABM). Alterations in the hexameric structure of the NC1 (α3 (IV)), allows novel epitopes to be exposed and an immune response to develop, with subsequent linear antibody deposition along the GBM, leading to a crescentic glomerulonephritis. Positive anti-GBM antibodies are assumed to be pathogenic and capable of binding GBM in vivo, especially in the context of rapidly progressive glomerulonephritis.

We have investigated patients with circulating anti-GBM antibodies, reactive to α3 (IV) and human GBM by immunoassays and Western blotting respectively, with focal necrotising crescentic glomerulonephritis but no linear GBM antibody deposition on immunohistochemistry. Three out of four were also ANCA positive. Despite not binding native GBM, patients’ sera showed linear binding to primate glomeruli by indirect immunofluorescence, in the 2 cases tested. Following treatment, significant improvements in kidney function were found in 3/4 patients.

**Case presentation:**

We present four patients with crescentic glomerulonephritis and circulating anti-GBM antibodies, but no glomerular binding.

**Conclusions:**

These novel findings, demonstrate that in some patients anti-GBM antibodies may not bind their own GBM. This has important implications for clinical diagnosis, suggesting that histological confirmation of kidney injury by anti-GBM antibodies should be obtained, as non-binding GBM antibodies may be associated with significant renal recovery.

**Electronic supplementary material:**

The online version of this article (10.1186/s12882-018-1027-x) contains supplementary material, which is available to authorized users.

## Background

Anti-GBM antibodies, are highly specific and sensitive for Goodpasture’s (anti-GBM) disease [1]. Antibodies are generally directed against the NC1 domain of the alpha 3 chain of type IV collagen(α3 (IV)) and less commonly, toward the α 4(IV) or α 5(IV) chains, which together form a triple helical structure in glomerular and alveolar basement membranes. Anti-GBM disease typically presents with a rapidly progressive crescentic glomerulonephritis (CGN) and pulmonary haemorrhage in approximately half of the patients. Despite the high specificity and sensitivity of the antibody tests, ‘false’ positive’ tests are reported in patients with polyclonal immunoglobulin responses following viral infections or drugs, but without CGN [2–4]. The gold standard for diagnosis is the kidney biopsy, demonstrating diffuse CGN with linear immunoglobulin staining along the GBM. Linear GBM immunoglobulin binding is also found in the elderly and in diabetic patients, although not in the context of CGN possibly due to changes in glycation patterns of the GBM antigens [5]. Additionally, atypical cases of anti-GBM disease have been reported, such as patients who lack circulating antibodies, detected using conventional assays, but with linear glomerular deposition on biopsy [6, 7], in some cases without CGN, in others with mesangial proliferative or endocapillary glomerulonephritis [7]. Circulating anti-GBM antibodies are detected in approximately 5–10% of sera from patients with anti-neutrophil cytoplasm antibody(ANCA) positive vasculitis [1], in some at lower titre than in patients with Goodpasture’s disease [1]. Experimental models have shown that low dose anti-GBM antibodies, that themselves do not induce CGN, can synergise with ANCA to augment glomerulonephritis, without development of linear anti-GBM staining [8, 9]. The synergy is in part due to increased production of glomerular cytokines and chemokines, as well as glomerular neutrophil activation. Here we present a cohort of four patients with circulating anti-GBM antibodies, two with high titre, and three with low level ANCA, all with CGN and no evidence of linear anti-GBM staining by immunohistochemistry or immunofluorescence. In all cases, sera bound recombinant α3 (IV), and two that were tested bound collagenase solubilised human GBM, while two also bound primate glomeruli. These data suggest that not all anti-GBM antibodies have similar pathogenic potential because some cannot bind their host GBM under normal circumstances.

### Case presentation

#### Case 1

A 66 year old Caucasian woman, was admitted to hospital with malaise, macroscopic haematuria and a petechial rash on both thighs. Her past medical history included seronegative rheumatoid arthritis and hypertension. Her kidney function at that point was normal, with a creatinine of 73 μmol/l (eGFR > 60 mls/min/1.73m^2^, MDRD formula), and there was no proteinuria. Investigations revealed negative ANA, ANCA, anti-GBM antibodies as well as hepatitis B and C serology. A kidney biopsy showed mild focal tubular and interstitial scarring, suggestive of modest chronic ischaemic damage, but no significant glomerular lesion and negative immunoperoxidase staining on formalin fixed tissue for all immunoproteins. Rapid resolution of the rash was seen following a course of high-dose prednisolone.

**Fig. 1:**
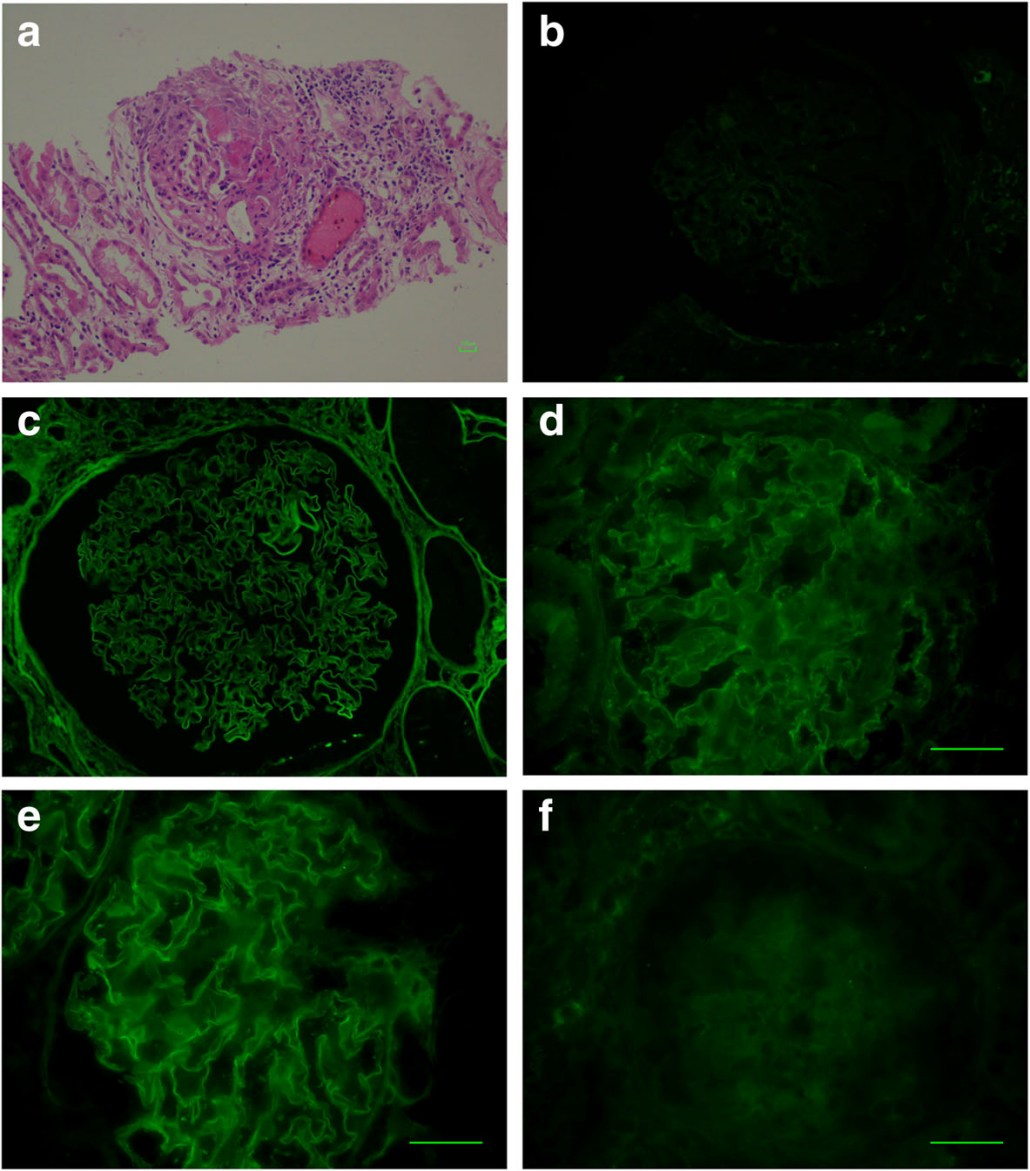
Kidney biopsy section from Patient 1 showing (**a**) pauci-immune focal necrotising glomerulonephritis (H and E, × 400) and (**b**) negative immunofluorescence for IgG (× 400); (**c**) A positive control showing linear IgG staining at identical exposure; (**d**) Indirect immunofluorescence staining of patient sera on primate kidney; (**e**) positive control indirect immunofluorescence and (**f**) negative control indirect immunofluorescence. Positive anti-glomerular basement membrane staining for IgG in a linear fashion was seen using the patient’s serum (× 400)

Four months following the discontinuation of prednisolone and after a flu like illness, the rash recurred, along with macroscopic haematuria, malaise and anorexia. On admission, she had a blood pressure of 162/90 and a purpuric rash over both thighs. Creatinine had risen to 241 μmol/L (eGFR 18 mls/min/1.73m^2^). Haemoglobin 10.9 g/dL, CRP 138 mg/L. Urine protein: creatinine ratio (PCR) was elevated at 150 mg/mmol; Tests revealed negative ANCA, ANA and rheumatoid factor, normal levels of immunoglobulins and complement. No anti-GBM antibody was obtained at this time. A repeat kidney biopsy demonstrated a severe, acute crescentic pauci-immune glomerulonephritis, with evidence of moderate chronic kidney damage. 13/22 glomeruli showed evidence of vasculitic lesions, 3 were globally sclerosed and 6 were normal. Immunoperoxidase and immunofluorescence were negative for IgG, IgM and IgA and C3 (Fig. 1).

Treatment with intravenous methylprednisolone and cyclophosphamide was commenced. An anti-GBM antibody titre, obtained 2 weeks post-discharge, was significantly elevated at 359 IU/ml (NR 0–10, ELiA, Phadia systems, Thermo Scientific). This elevation was confirmed on repeat testing. 14 cycles of plasma exchange were performed with 6 doses of pulsed cyclophosphamide. At four months creatinine was 130 μmol/l and at 4 years 105 μmol/l (eGFR 37 and 47 mls/min respectively)while ANCA and anti-GBM have remained negative (Table 1).

**Table 1:** Summary of patients with circulating anti-GBM antibodies without linear deposition

Age	Gender	Anti-GBM titre at presentation (NR < 10)	ANCA/Subtype/ titre	Peak creatinine at presentation (μmol/l)	Last follow up creatinine (μmol/l)	Time to anti-GBM negativity/months	Follow up/months	Pulmonary haemorrhage?	Treatment
66	F	359	Negative	271	105	4	50	N	Prednisolone Methylprednisolone Cyclophosphamide Plasma exchange
70	F	200	Yes/MPO/20	809	HD	4	24	N	Methylprednisolone Prednisolone Plasma exchange Cyclophosphamide Immunoadsorption Rituximab
79	F	28	Yes/MPO/33	446	199	2	18	N	Methylprednisolone Prednisolone Rituximab Cyclophosphamide
64	M	33	Yes/MPO/60	666	127	0.9	21	N	Methylprednisolone Plasma exchange Prednisolone Cyclophosphamide Mycophenolate mofetil

#### Case 2

A 70 year old previously healthy Caucasian woman presented with lethargy, anorexia, nausea, vomiting, and a two kilogram weight loss over the course of a fortnight.

**Fig. 2:**
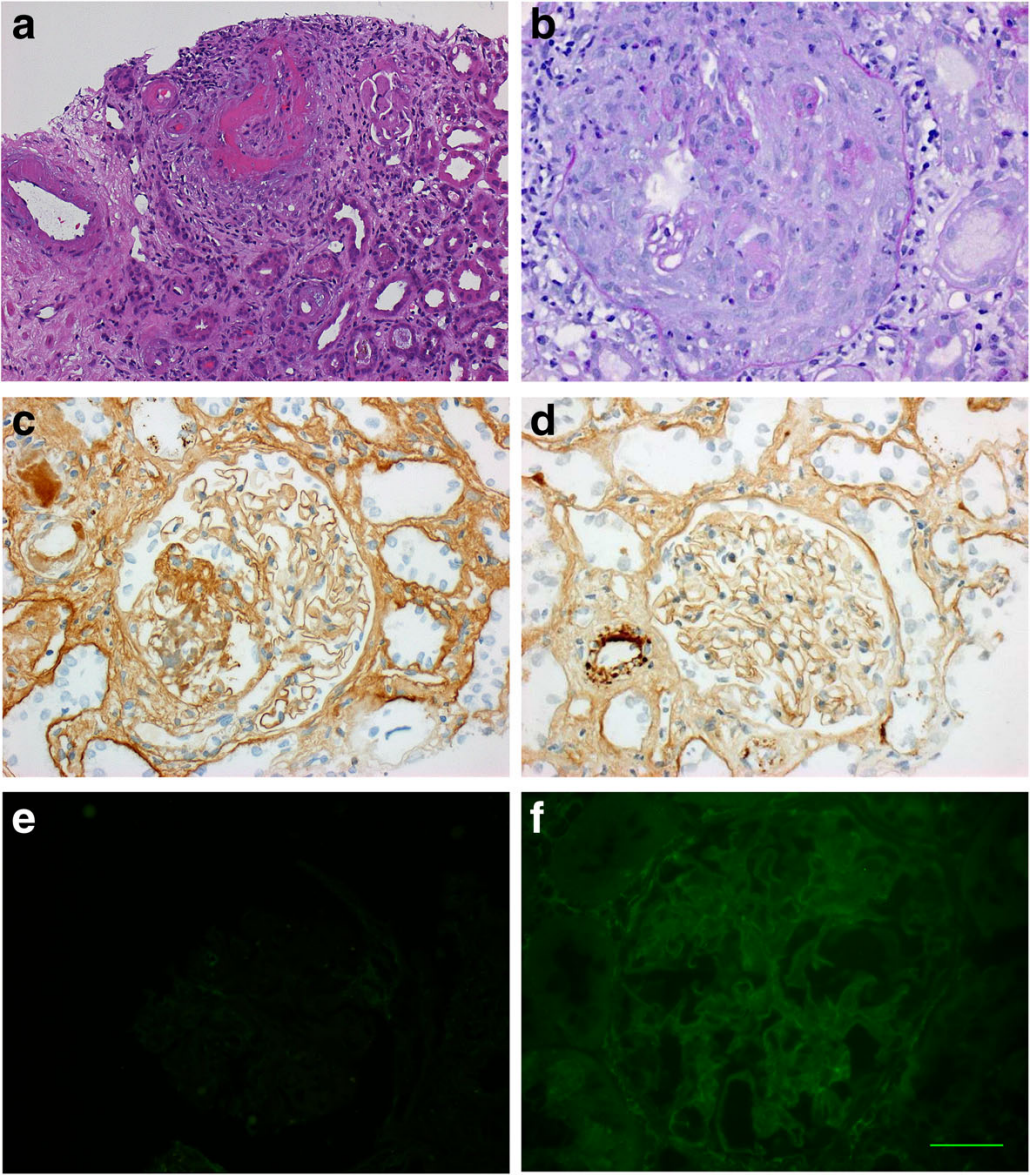
Kidney biopsy sections from patient 2 showing (**a**) focal necrotising crescentic glomerulonephritis (H and E × 200); (**b**) PAS stain of a cellular crescent (× 400); Immunoperoxidase stains for (**c**) IgG and (**d**) C3; (**e**) Immunofluoresence for IgG (all × 400); (**f**) Indirect immunofluorescence staining of patient sera on primate kidney

Investigations showed an elevated serum creatinine of 477 μmol/L (eGFR 9 ml/min, CKD-EPI formula), with a previous creatinine of 68 μmol/L (eGFR 91 ml/min) 3 months earlier. The blood pressure was 180/100 mmHg, other physical findings were normal. Urinalysis revealed blood and protein, while microscopy confirmed erythrocytes, leukocytes, dysmorphic red cells, but no red cell casts. Serology showed positive MPO-ANCA with a titre of 20 IU/ml (NR 0–5) and high titre anti-GBM antibody titre > 200 IU/ml (NR 0–20; Alegria ELISA, Orgentec) and 475 U/ml (using EliA Phadia assay), complement levels were normal. Kidney function deteriorated over the next few days, with creatinine reaching 809 μmol/l (eGFR 5 ml/min). Kidney biopsy showed diffuse extracapillary necrotizing glomerulonephritis, interstitial inflammation and leucocytoclastic necrotizing vasculitis. Immunohistochemistry on formalin fixed tissue showed no immune deposits along the GBM (Fig. 2). She was treated with methylprednisolone pulses, followed by oral prednisolone. Haemodialysis and 7 plasma exchanges were also initiated followed by intravenous cyclophosphamide. Although urine output improved, she remained dialysis dependent and her repeat anti-GBM antibody remained strongly positive. She therefore underwent 3 sessions of immunoabsoprtion on a protein A column which led to a drop in anti-GBM titer from > 200 IU/l to 25 IU/l. Due to severe leucocytopenia with consecutive pneumonia following the first dose of cyclophosphamide, she was switched to weekly rituximab, which was also stopped after the third dose following further infections. Anti-GBM antibodies were negative at 4 months. She has remained dialysis dependent 24 months later (Table 1).

#### Indirect immunofluorescence and western blotting

In order to test whether the anti-GBM antibodies were capable of binding intact glomeruli, we used a validated anti-GBM assay based on staining of monkey glomeruli (Eurimmun GBM assay FA1250). Sections were incubated with serum diluted 1:10 in PBS-Tween according to manufacturer’s instructions. As expected the positive control samples stained with a linear pattern along the GBM, while sera from patients 1 and 2 stained with a similar but less intense pattern, suggesting that they were capable of binding the exposed epitopes in a native glomerulus, but not their own GBM (Figs. 1 and 2). Western blotting using collagenase solubilised human GBM showed positivity with 2/4 samples available (Additional file 1: Figure S1), confirming the ability of the antibodies to bind human collagenase solubilised GBM.

#### Case 3

A 64 year old man presented to a local community hospital with a 5 week history of dry cough and fevers which has persisted despite 2 courses of oral antibiotics. There was a 10 year history of unclassified arthralgia. Presenting creatinine was 682 μmol/L Urinalysis: 3 + blood and protein. Urine microscopy revealed granular casts with greater than 200 red cells/ cu.mm. Urinary protein: creatinine (uPCR) ratio was 133 mg/mmol. Physical examination was unremarkable, but BP 186/74 on admission. MPO-ANCA titre was 60 IU/ml (NR < 5) and a positive anti-GBM titre of 33 IU/ml (NR < 10, ELiA, Phadia systems). Complement levels normal. Renal biopsy revealed crescentic glomerulonephritis in 80% of glomeruli. However, no glomerular deposition of immunoglobulin or complement was seen on formalin fixed tissue (Additional file 2: Figure S2). The patient was treated with plasma exchange, pulsed methylprednisolone, followed by oral prednisolone and six pulses of intravenous cyclophosphamide. Maintenance therapy was with a reducing course of prednisolone and mycophenolate mofetil, due to azathioprine intolerance. At 21 months of follow up serum creatinine was 127 μmol/L, and both MPO-ANCA and anti-GBM were negative (Table 1).

#### Case 4

A 79 year old woman presented to a local hospital with a 2 week history of epistaxis, arthralgia, weight loss and anorexia. She gave a history of previous recurrent iritis, with the last episode occurring 2 year prior to her presentation. Physical examination was unremarkable apart from peripheral oedema. Presenting creatinine was 430 μmol/L. Urine dipstick revealed + 3 blood and + 2 protein, and uPCR 259 mmol/mg. MPO-ANCA titer was 33 IU/ml(NR < 5) and an anti-GBM titre of 28 IU/ml(NR < 10, ELiA, Phadia systems). Renal biopsy revealed a pauci-immune crescentic glomerulonephritis, with no staining for IgG, IgA or IgM on formalin fixed tissue. There was some chronic parenchymal damage (Additional file 2: Figure S2). Treatment was with methylprednisolone, a short course of oral prednisolone, rituximab and six pulses of intravenous cyclophosphamide. She was also intolerant of maintenance azathioprine. Her MPO-ANCA and anti-GBM titres remained < 1 IU/ml and her creatinine was 199 μmol/L after 18 months’ follow-up (Table 1).

## Discussion and conclusions

In this case series we present four patients with a pauci-immune rapidly progressive glomerulonephritis with anti-GBM antibodies, two with high titre, but without linear deposition of antibody on the basement membrane. The relevance of the anti-GBM antibodies in such patients has not been focussed on previously. While pauci-immune vasculitis lacking ANCA is a well-recognised phenomenon representing between 27 and 38% of pauci-immune GN [10], none have been reported with circulating anti-GBM antibodies.

All patients presented with non-specific prodromal symptoms and significant kidney injury. In three out of the four there was a substantial improvement in kidney function following therapy, while one patient remained dialysis dependant.

Anti-GBM antibodies are considered diagnostic for Goodpasture’s disease and it has been suggested that the level of anti-GBM antibodies correlates with long term prognosis [11]. In addition, there are case reports of low levels of anti-GBM antibodies being associated with an indolent disease process [12]. Anti-GBM antibodies are found in co-existence with ANCA [13] in up to 5% of ANCA positive subjects and in one series all of the biopsied patients showed evidence of linear antibody deposition [13]. It is notable that 18% of these double positive patients reported were not biopsied, meaning that some may have had lacked linear staining on the GBM as we have described. Interestingly, none of the patients described with creatinine levels > 500 μmol/ recovered kidney function following immunosuppression with or without adjunctive plasma exchange. This is in contrast with our patients in whom kidney function recovery was found in the majority.

All of our patients had binding to recombinant alpha3 (IV) collagen, and in both tested to collagenase solubilised human GBM, demonstrating that these antibodies were similar with those found in typical anti-GBM disease. In addition, in 2 they bound primate kidney in a linear fashion- another standardised assay for detecting anti-GBM antibodies. Previous reports have shown that in double positive(ANCA and anti-GBM antibody) patients there may be a broader specificity to the anti-GBM antibodies and that titres are lower than in anti-GBM disease alone [14], however in two of our subjects high anti-GBM titres were found.

The reason for the lack of linear binding in vivo is intriguing, but the ability of the antibodies to bind fixed primate kidney and collagenase digested human GBM, suggests that one possible reason is that the patients do not express the required epitope to allow antibody binding. In the native GBM, the cryptic nature of the antigenic epitopes is related to the quaternary structure of the of alpha 3(IV) hexamers. Whilst autoantibodies can bind some monomer containing hexamers (M-hexamers), they cannot bind those containing both monomers and dimers (D-hexamers) [15]. Alteration in the proportion of M and D hexamers in the ABM is one possible explanation for the variation in pulmonary haemorrhage seen in some patients with Goodpasture’s disease [16] and there may be a lack of exposure of the appropriate M hexamers in our subjects’ GBM, preventing antibody binding in vivo. This may also explain the cases of anti-GBM antibody presenting with only pulmonary haemorrhage [17]. Interestingly, in both of the patients tested, binding to whole collagenase solubilised GBM demonstrated binding to dimers only and not monomers (Additional file 1: Figure S1). However, this raises the question as to how the immune response to alpha3 (IV) was generated initially, which may have involved some degradation of GBM following the pauci-immune glomerular insult, or indeed exposure of epitopes from the ABM. An experimental model in which T cell mediated anti-GBM damage is induced without deposited antibodies has been described, which may provide a potential disease mechanism [18]. Finally, there is the possibility that the antigenic epitope or IgG were not exposed by the standard histological processing following collection of the sample into formalin, although they were all treated in conventional ways, and both immunofluorescence and immunoperoxidase detection of linear IgG were negative (Figs. 1-2 and Additional file 2: Figure S2), while IgG staining of tubular protein droplets and interstitial cells was observed making a processing issue less likely (Additional file 3: Figure S3). These findings have implications for clinical practice, suggesting we should obtain histological confirmation of disease so that patients can be appropriately counselled and managed.

## Additional files

Additional file 1: **Figure S1.** Western blot using whole human collagenase solubilized GBM using positive control (patient with circulating anti-GBM antibodies with linear glomerular binding and crescentic nephritis) and patients 3 and 4, demonstrating binding to alpha 3 dimers and monomers in the positive control but only dimers in patients 3 and 4. (PDF 18 kb)

Additional file 2: **Figure S2.** Renal biopsy sections from patients 3 and 4. (TIF 1290 kb)

Additional file 3: **Figure S3.** IgG staining of biopsy from patient 4 showing protein droplets in renal tubular cells staining positive for IgG and immunofluorescence of biopsy from patient 2 showing IgG staining of interstitial cells. (TIF 869 kb)

## Abbreviations

ANCA: Anti-neutrophil cytoplasm antibody
CGN: Crescentic glomerulonephritis
GBM: Glomerular basement membrane disease
NC1: Non collagenous domain 1
