# Supplementary figures and images for: Crescentic glomerulonephritis with anti-GBM antibody but no glomerular deposition
Source: BMC Nephrol. 2018 Sep 12;19:228. doi: 10.1186/s12882-018-1027-x (PMC6136232; doi:10.1186/s12882-018-1027-x)

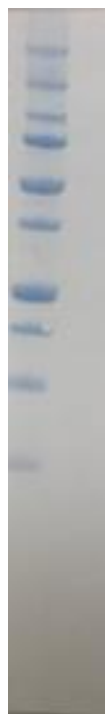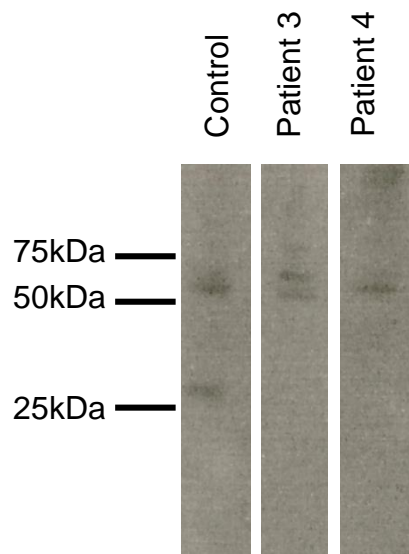

Supplement: Supplementary file 1 — Figure S1. Western blot using whole human collagenase solubilized GBM using positive control (patient with circulating anti-GBM antibodies with linear glomerular binding and crescentic nephritis) and patients 3 and 4, demonstrating binding to alpha 3 dimers and monomers in the positive control but only dimers in patients 3 and 4. (PDF 18 kb) [file 12882_2018_1027_MOESM1_ESM.pdf]

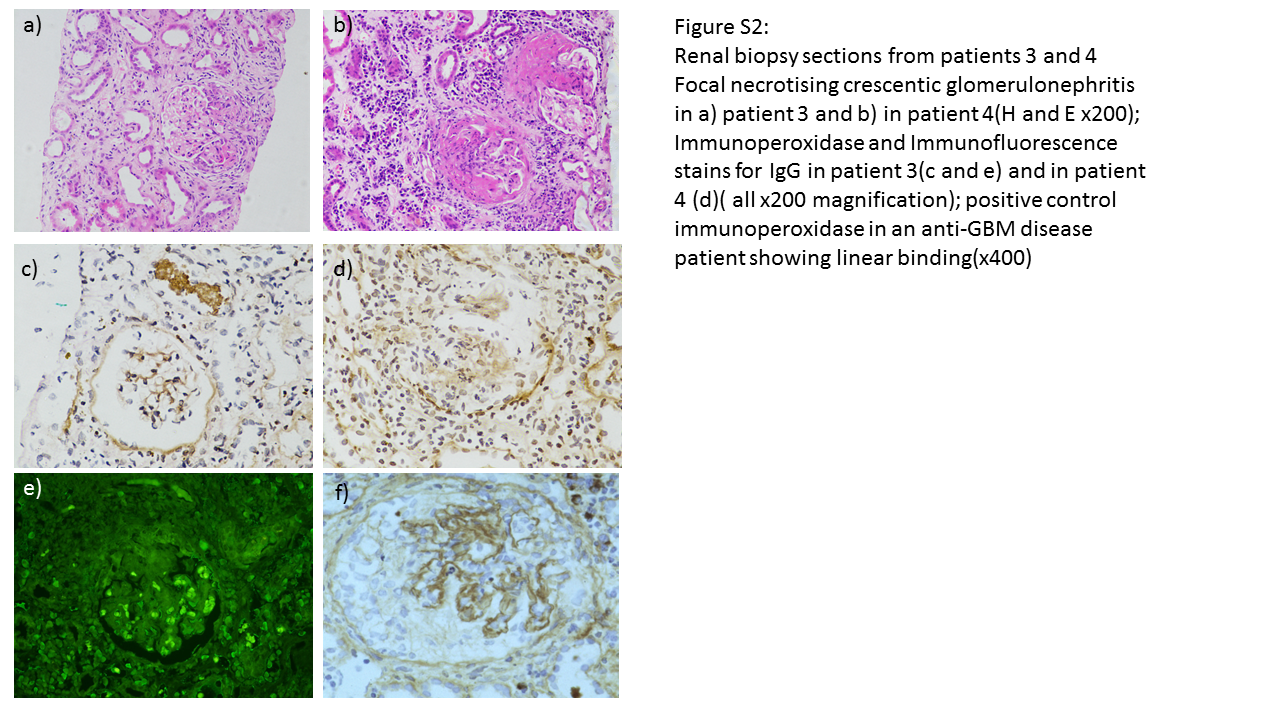

Supplement: Supplementary file 2 — Figure S2. Renal biopsy sections from patients 3 and 4. (TIF 1290 kb) [file 12882_2018_1027_MOESM2_ESM.tif]

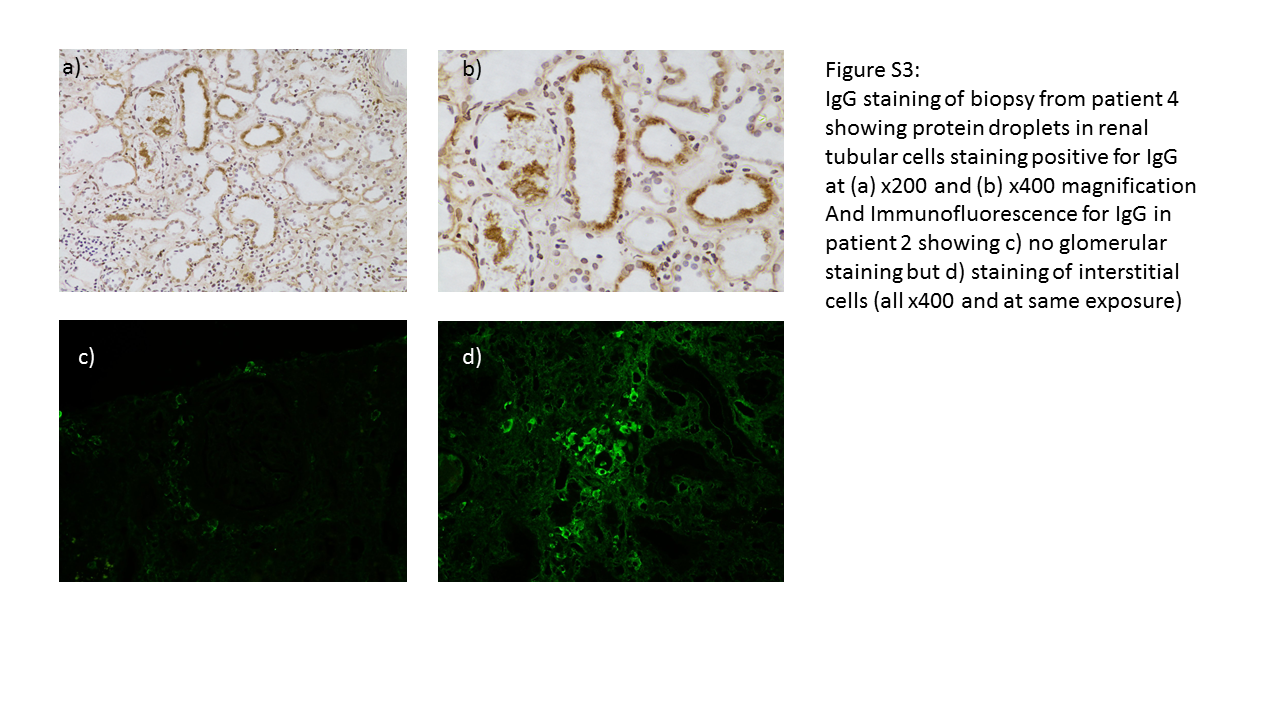

Supplement: Supplementary file 3 — Figure S3. IgG staining of biopsy from patient 4 showing protein droplets in renal tubular cells staining positive for IgG and immunofluorescence of biopsy from patient 2 showing IgG staining of interstitial cells. (TIF 869 kb) [file 12882_2018_1027_MOESM3_ESM.tif]
